# Living on the edge: substrate competition explains loss of robustness in mitochondrial fatty-acid oxidation disorders

**DOI:** 10.1186/s12915-016-0327-5

**Published:** 2016-12-07

**Authors:** Karen van Eunen, Catharina M. L. Volker-Touw, Albert Gerding, Aycha Bleeker, Justina C. Wolters, Willemijn J. van Rijt, Anne-Claire M. F. Martines, Klary E. Niezen-Koning, Rebecca M. Heiner, Hjalmar Permentier, Albert K. Groen, Dirk-Jan Reijngoud, Terry G. J. Derks, Barbara M. Bakker

**Affiliations:** 1 Department of Pediatrics, University of Groningen, University Medical Center Groningen, Hanzeplein 1, 9713 GZ Groningen, The Netherlands; 2 Department of Laboratory Medicine, University of Groningen, University Medical Center Groningen, Hanzeplein 1, 9713 GZ Groningen, The Netherlands; 3 Analytical Biochemistry and Interfaculty Mass Spectrometry Center, University of Groningen, A. Deusinglaan 1, 9713 AV Groningen, The Netherlands; 4 Section of Metabolic Diseases, Beatrix Children’s Hospital, University of Groningen, University Medical Center Groningen, Hanzeplein 1, 9713 GZ Groningen, The Netherlands; 5 Top Institute for Food and Nutrition, Nieuwe Kanaal 9A, 7609 PA Wageningen, The Netherlands; 6 Systems Biology Center for Energy Metabolism and Aging, University of Groningen, University Medical Center Groningen, A. Deusinglaan 1, 9713 AV Groningen, The Netherlands; 7 Present address: Department of Medical Genetics, University Medical Center Utrecht, Utrecht, The Netherlands; 8 PO Box 196, Internal ZIP code EA12, NL-9700 AD Groningen, The Netherlands

**Keywords:** Medium-chain acyl-CoA dehydrogenase deficiency, Multiple acyl-CoA dehydrogenase deficiency, Mitochondrial fatty-acid oxidation, Systems medicine, Kinetic modeling

## Abstract

**Background:**

Defects in genes involved in mitochondrial fatty-acid oxidation (mFAO) reduce the ability of patients to cope with metabolic challenges. mFAO enzymes accept multiple substrates of different chain length, leading to molecular competition among the substrates. Here, we combined computational modeling with quantitative mouse and patient data to investigate whether substrate competition affects pathway robustness in mFAO disorders.

**Results:**

First, we used comprehensive biochemical analyses of wild-type mice and mice deficient for medium-chain acyl-CoA dehydrogenase (MCAD) to parameterize a detailed computational model of mFAO. Model simulations predicted that MCAD deficiency would have no effect on the pathway flux at low concentrations of the mFAO substrate palmitoyl-CoA. However, high concentrations of palmitoyl-CoA would induce a decline in flux and an accumulation of intermediate metabolites. We proved computationally that the predicted overload behavior was due to substrate competition in the pathway. Second, to study the clinical relevance of this mechanism, we used patients’ metabolite profiles and generated a humanized version of the computational model. While molecular competition did not affect the plasma metabolite profiles during MCAD deficiency, it was a key factor in explaining the characteristic acylcarnitine profiles of multiple acyl-CoA dehydrogenase deficient patients. The patient-specific computational models allowed us to predict the severity of the disease phenotype, providing a proof of principle for the systems medicine approach.

**Conclusion:**

We conclude that substrate competition is at the basis of the physiology seen in patients with mFAO disorders, a finding that may explain why these patients run a risk of a life-threatening metabolic catastrophe.

**Electronic supplementary material:**

The online version of this article (doi:10.1186/s12915-016-0327-5) contains supplementary material, which is available to authorized users.

## Background

Mitochondrial fatty-acid oxidation (mFAO) is essential for providing energy during periods of fasting or other metabolic stress. In humans, more than 15 different inborn errors of metabolism have been described in this pathway. These genetic disorders affect organs such as the liver, heart and skeletal muscle [1], and together they constitute a large group of individually rare diseases [2]. The fact that affected patients show genetic and phenotypic heterogeneity calls for treatment tailored to the individual, i.e., personalized medicine. Some patients, such as those with medium acyl-CoA dehydrogenase (MCAD) deficiency, have few or no symptoms under normal circumstances [3–5], these patients can oxidize fatty acids completely and at a normal rate [6]. This may be because the role of MCAD is taken over by isoenzymes such as short-chain acyl-CoA dehydrogenase (SCAD) and very-long-chain acyl-CoA dehydrogenase (VLCAD). In contrast, other mFAO disorders have a severe neonatal phenotype, as is the case in VLCAD deficiency and in severe cases of multiple acyl-CoA dehydrogenase deficiency (MADD).

The clinical manifestations of mFAO disorders can be aggravated by fasting, exposure to cold, or exercise [7, 8]. Such circumstances may then lead to sudden death, biochemically associated with hypoketotic hypoglycemia, even in patients with the apparently mild MCAD deficiency. Thus, the deficiency of a single enzyme that is not essential under normal circumstances reduces the robustness of the metabolic network, rendering it less able to cope with metabolic challenges. The mechanism that underlies this sudden crisis is not well understood. A recent study in mice lacking the long-chain acyl-CoA dehydrogenase enzyme (*Lcad* ^*–/–*^) showed low blood-glucose levels during fasting to be related to the supply of amino-acid precursors for hepatic glucose production [9]. Yet, the link between this shortage of amino acids and the primary enzyme defect in mFAO is still unclear. This illustrates that, although the enzymes involved in mFAO are well known, our understanding of the regulation of this pathway is far from complete. Further insights into mFAO functioning will not only help to improve the treatment of patients with inherited mFAO disorders, but should also contribute to a better understanding of how the mFAO pathway is involved in multifactorial and age-related diseases, such as obesity and type 2 diabetes [10].

The metabolism of a healthy individual is exceptionally robust with respect to perturbations in genetics, nutrition and workload, in the sense that key metabolic functions are normally unaffected by such perturbations [11]. In particular, the levels of blood glucose and cellular ATP are maintained within a narrow range. This is why mammals shift from carbohydrate oxidation to mFAO during fasting. The oxidation of fatty acids then provides a reliable supply of ATP for glucose production by the liver. This key function of the liver often fails unpredictably in mFAO disorders, leading to a sudden drop in glucose levels, triggered, for example, by fasting during intercurrent infections [12]. This sensitivity of individuals with mFAO disorders to external perturbations means that this group of diseases is particularly suitable for the study of metabolic robustness.

**Fig. 1:**
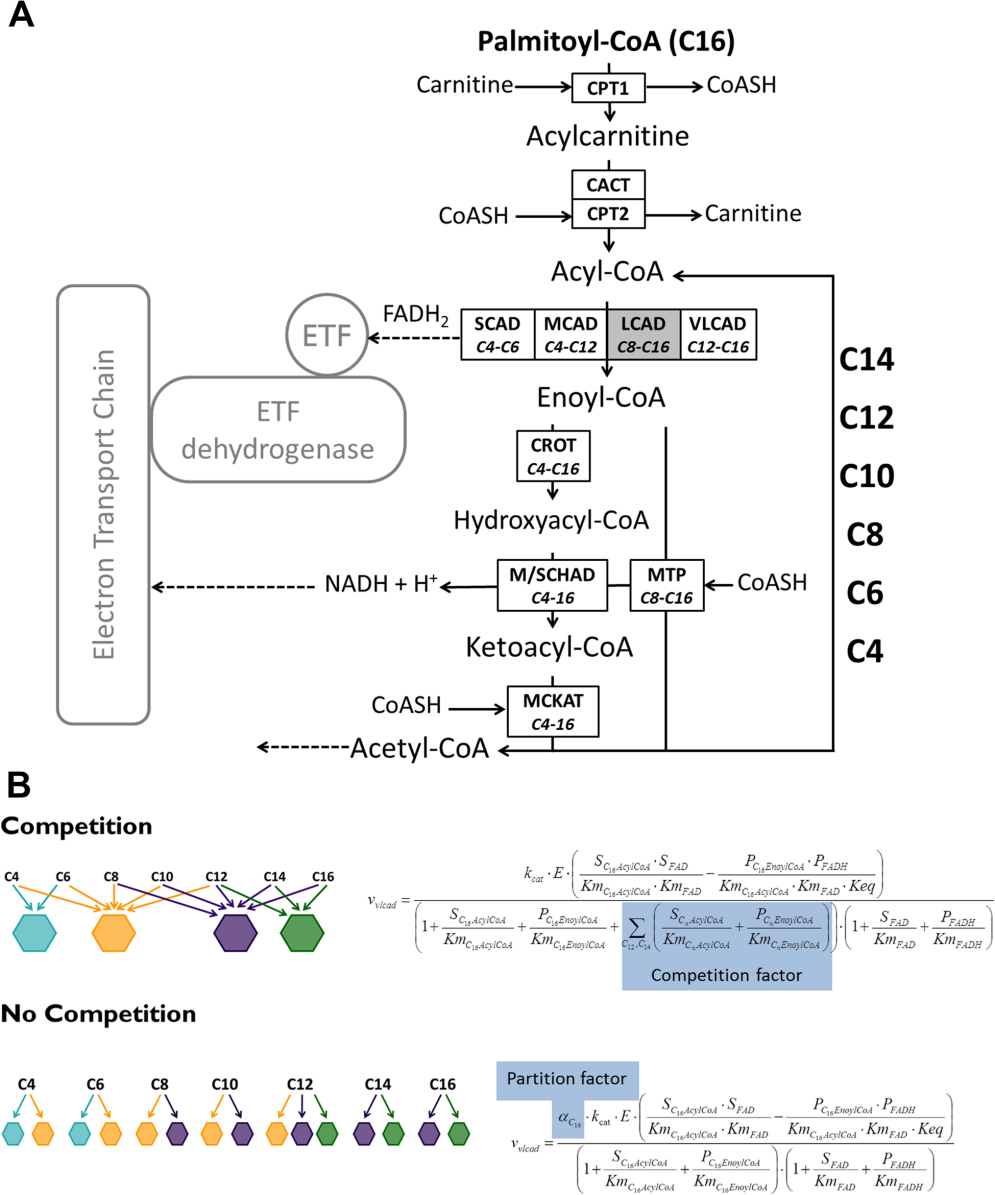
Modeling of mFAO in wild-type and MCAD-knockout mouse liver. **a** Schematic representation of fatty acid β-oxidation. Reactions in black are part of the computational model, reactions in grey are not in the model, but are discussed in the text and dashed arrows are sink reactions. **b** Examples of rate equations used for the model with and without competition. *CPT1* carnitine palmitoyltransferase 1, *CACT* carnitine acylcarnitine translocase, *CPT2* carnitine palmitoyltransferase 2, *SCAD* short-chain acyl-CoA dehydrogenase, *MCAD* medium-chain acyl-CoA dehydrogenase, *LCAD* long-chain acyl-CoA dehydrogenase (not present in human mFAO (grey)), *VLCAD* very-long-chain acyl-CoA dehydrogenase, *CROT* crotonase, *M/SCHAD* medium/short-chain hydroxyacyl-CoA dehydrogenase, *MCKAT* medium-chain ketoacyl-CoA thiolase, *MTP* mitochondrial trifunctional protein, *ETF* electron transfer flavoprotein

Since robustness is a dynamic property of a metabolic network, its description cannot rely exclusively on a static representation of data, but requires kinetic computational modeling. Like polymers, fatty acids are degraded in repetitive cycles and until now the systemic implications of such a pathway structure have hardly been explored. The pathway of mFAO in the liver is illustrated schematically in Fig. 1a. In each oxidation cycle, an acyl-CoA ester (the activated form of a fatty acid) is shortened by two carbon atoms and the product becomes the substrate for the next cycle. In rodents, the first step in mFAO is catalyzed by four isoenzymes (VLCAD, LCAD, MCAD and SCAD), which together cover CoA esters with chain lengths ranging from 4 to 16 carbon atoms. Substrates of different chain lengths therefore compete for common enzymes. Since each enzyme molecule can only bind one substrate molecule at a time, the alternative substrates for the same enzyme act as competitive inhibitors. MCAD, for instance, catalyzes the dehydrogenation of acyl-CoA esters of 4 to 12 carbon atoms (Fig. 1a). The dehydrogenation of any of these substrates is competitively inhibited by binding of all the other substrates and products. This principle applies to all enzymes in the pathway, generating a large number of feedforward and feedback inhibition loops in the pathway. The absence of the one enzyme–one reaction relationship and the resulting substrate competition has a major impact on the systemic properties of lipid and polymer metabolism in general. The mFAO is not the only cellular pathway where substrate competition takes place; this is a general feature of lipid metabolism [13]. Moreover, mRNA species are also known to compete for ribosomes and enzymes in both lipid and glycogen metabolism are known to typically accept substrates of various chain lengths and branching types. The few studies that have investigated polymer metabolism quantitatively – by applying thermodynamics, kinetic modeling, and control analysis – have related it to enhanced robustness on the one hand [14] as well as ultra-sensitivity to environmental challenges on the other [15, 16].

In this study, we took a systems-biology approach to investigate whether and how substrate competition is involved in the characteristic features of mFAO disorders, and particularly in the loss of metabolic robustness in these patients. We made use of our recently published dynamic computational model of mFAO, which describes substrate competition in terms of detailed kinetic equations for each of the reactions of Fig. 1a [16]. This model is based on an extensive collection of enzyme kinetic data from purified enzymes. As independent validation, we showed that measured time courses of acylcarnitines could be predicted quite accurately by model simulations [16]. We combined this computational model with the MCAD knockout (KO) mouse, which is reported to mimic characteristics typical of MCAD-deficient patients such as elevated serum levels of medium-chain acylcarnitines, cold intolerance after fasting and high neonatal mortality [17]. We also made use of diagnostic acylcarnitine profiles of patients to study the physiological and clinical relevance of substrate competition. For reasons that will be explained later, the profiles of MADD patients proved particularly insightful.

Here we provide evidence that (1) substrate competition in mFAO – a mechanism inherent to the repetitive metabolism of fatty acids – renders the pathway vulnerable to substrate overload, particularly in the absence of MCAD; (2) this substrate competition is physiologically relevant since it is a key factor in explaining the patient-specific acylcarnitine profiles of MADD patients; and (3) it is clinically relevant since a computational model that included substrate competition was able to explain the severity of the patients’ symptoms from their acylcarnitine profiles, while a similar model without competition could not.

## Results

To explore the mechanism underlying loss of metabolic robustness in MCAD deficiency, our first aim was to adapt the existing computational model of mFAO in rat liver. To this end, we collected quantitative data from the livers of MCAD-KO and wild-type mice.

### MCAD-KO mouse characteristics

The MCAD-KO mouse created by Tolwani et al. [17] was backcrossed from a mixed 129P2xC57BL6/J background to a C57BL6/J background. The basic physiological characteristics of the newly obtained strain can be found in Additional file 1: Table S1 and Additional file 2: Figure S2A–E. The residual enzyme activity in liver homogenates of MCAD-KO mice, as measured with a clinical diagnostic assay for MCAD, was 9.3% of the wild-type activity (Additional file 2: Figure S2D). The activity was measured with phenylpropionyl-CoA as a supposedly MCAD-specific substrate. This percentage is similar to that found previously in the 129P2xC57BL6/J MCAD-KO strain [18]. Most likely, the residual activity in the KO mice originates from peroxisomal phenylpropionyl-CoA oxidation rather than from MCAD itself [19]. Bloodspots of MCAD-KO mice contained elevated medium-chain acylcarnitine levels (C6-C10 and C10:1) and an increased ratio of C8/C10 acylcarnitines (Additional file 2: Figure S2A, B). This metabolic profile is typical of MCAD-KO mice [17] and similar to that of patient profiles [20].

### Knockout of MCAD is not compensated for by increased capacity of other enzymes

**Fig. 2:**
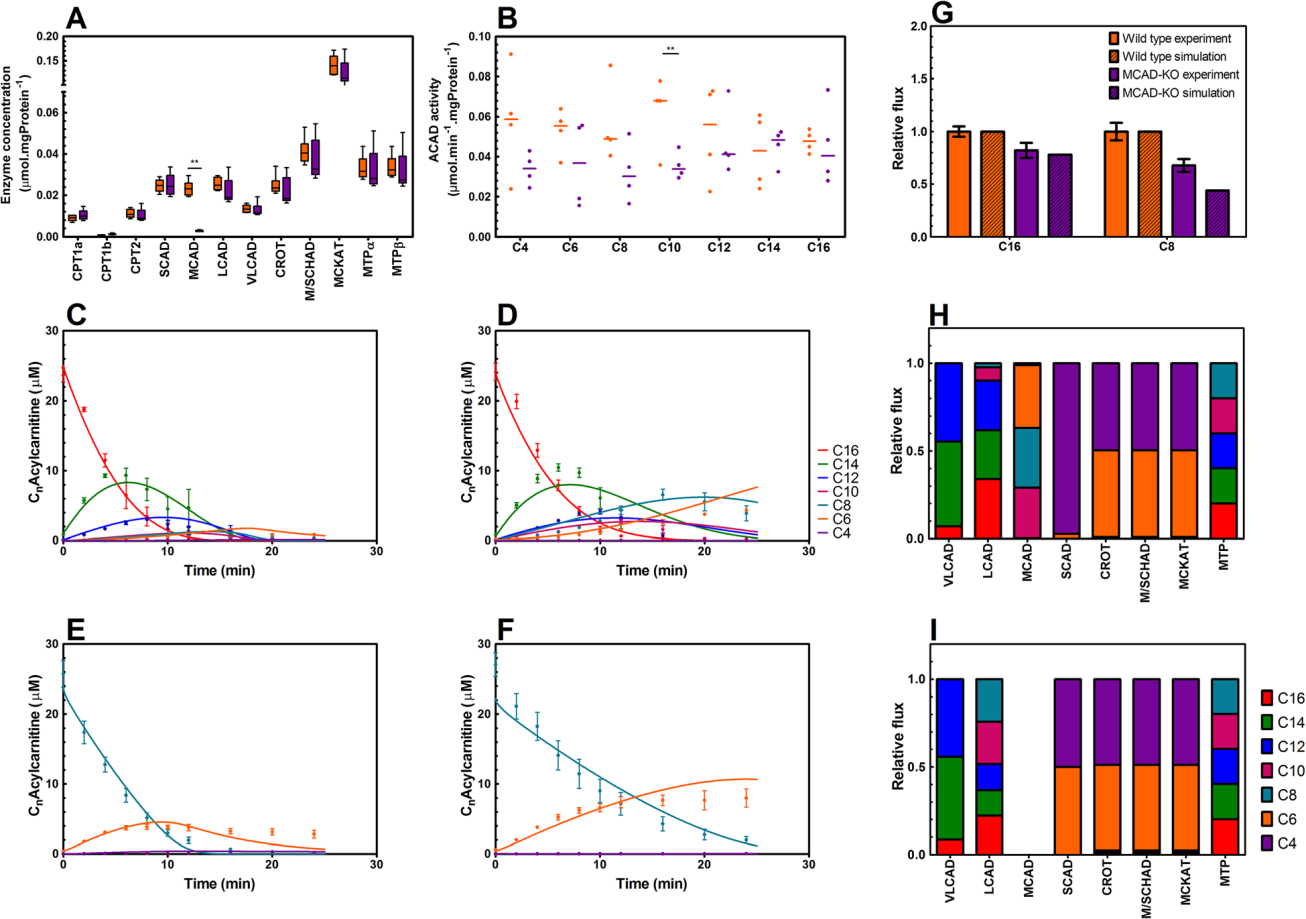
Experimental and simulation data of the mFAO model of wild-type and MCAD-knockout (KO) mouse liver. **a** Concentrations of mFAO enzymes per mg mitochondrial protein in wild-type (orange bars) and MCAD-KO (purple bars) mouse livers. Data represents median, the box extends from the 25^th^ to 75^th^ percentile and the whiskers extend from the minimum to the maximum value (n = 6). **b** Total acyl-CoA dehydrogenase (ACAD) activity for substrates of varying carbon chain lengths per mg mitochondrial protein measured in homogenate of isolated mitochondria in wild-type (orange) and MCAD-KO (purple) mouse livers. Data represents median and each individual data point (n = 4). Panels **c**–**f** show the dynamic profiles of the C_n_-acylcarnitines upon addition of the substrate palmitoylcarnitine (C16; panel **c** and **d**) and octanoylcarnitine (C8; panel **e** and **f**) to isolated mitochondria of wild-type livers (panel **c** and **e**) and MCAD-KO livers (panel **d** and **f**) in the presence of the uncoupler FCCP. Symbols represent the data measured (data represents mean ± SEM (n = 6)) and lines represent the simulation after parameter estimation. The color scheme indicated in panel **d** is similar for panels **c**–**f**. Panel **g** shows the relative change in maximum oxidation rate (flux) normalized to the rate of the wild-type, in experiments (oxygen consumption rate measured during 25 minutes in the presence of FCCP; data represents mean ± SEM (n = 6)) and in dynamic simulations (rate of NADH production by mFAO simulated over the same time period). Panels **h** and **i**: Distribution of steady-state flux across the various chain lengths for the mFAO enzymes simulated in the computational model of mouse liver mFAO, for wild-type (panel **h**) and MCAD-KO (panel **i**). *P < 0.05, **P < 0.01

Subsequently, we studied whether the knockout of MCAD was compensated for by altered expression of genes encoding other mFAO enzymes. As expected, MCAD was below the detection limit in the liver, both at mRNA and protein level. However, neither real-time PCR (Additional file 2: Figure S2F) nor quantitative, targeted proteomics (Fig. 2a) showed any significant differences between knockout and wild-type mice for any of the other transcripts or proteins involved in mFAO. Notably, the concentrations of the other acyl-CoA dehydrogenases (SCAD, LCAD and VLCAD) – which have overlapping substrate specificity with MCAD – were unchanged. Moreover, the enzyme activities of crotonase (CROT), medium/short-chain hydroxyacyl-CoA dehydrogenase (M/SCHAD) and medium-chain ketoacyl-CoA thiolase (MCKAT) for C4 substrates did not differ significantly between mitochondrial homogenates of MCAD-KO and wild-type mice (Additional file 2: Figure S2G).

We measured the total acyl-CoA dehydrogenase (ACAD) activity for substrates over the entire range of chain lengths from C4 to C16. In the MCAD-KO, enzyme activity was decreased for the short- and medium-chain acyl-CoAs (C4–C10), but unchanged for the longer chain lengths (Fig. 2b). Since C4–C10 acylcarnitines are substrates for MCAD, this result is consistent with loss of MCAD activity without compensation by covalent modifications of the other ACAD enzymes. Indeed, by deconvolution of the total acyl-CoA dehydrogenase activities for the various chain lengths, we could fit the specificity for the chain length as well as the *V*_*max*_ values of SCAD, LCAD and VLCAD to a single parameter set for MCAD-KO and wild-type mice (Additional file 3: Text S3).

Overall, the protein concentrations of mFAO enzymes measured did not suggest compensation for the knockout of MCAD activity, nor did we find an indication for compensation through changes in their specific activity.

### Conversion of the dynamic mFAO model from rat to mouse liver

To convert our previously constructed dynamic model of mFAO in rat liver [16] into one of mouse liver, we completed the above-described dataset by measuring the acylcarnitine concentrations with chain lengths C4–C16 over time. At time zero, palmitoylcarnitine or octanoylcarnitine was given to isolated liver mitochondria of wild-type and MCAD-KO mice (Fig. 2c–f, symbols). The knockout of MCAD led to increased levels of decanoyl- (C10), octanoyl- (C8) and hexanoylcarnitine (C6) and a reduced rate of octanoylcarnitine consumption.

The above-measured data on enzyme kinetics, including the parameter set for the acyl-CoA dehydrogenases, were directly incorporated into the model. Subsequently, additional parameter values were fitted to the acylcarnitine time courses. Since models with complex biochemical rate equations are typically underdetermined [21], we only fitted the parameters to which the acylcarnitine concentrations were most sensitive (see Methods and Additional file 4: Table S4 for rationale and estimated parameter values). The fitted model (Additional file 5: Model S5) described the experimental data accurately (Fig. 2c–f; symbols: experimental data; lines: model simulations).

The fluxes served as validation data, as they had not been used for parameter fitting. When expressed relative to wild-type, the oxidation of palmitoylcarnitine (C16) was not significantly reduced in the MCAD-KO, both in experiment and simulation (Fig. 2g). This was due to the fact that this flux was hardly controlled by MCAD at the timescale of the experiment (25 minutes) and the role of MCAD was effectively taken over by the other ACADs (Additional file 6: Table S6). The oxidation of octanoylcarnitine (C8), however, which directly feeds octanoyl-CoA into MCAD, was substantially reduced in the MCAD-KO compared to the wild-type, in agreement with the higher flux control by MCAD under this condition (Additional file 6: Table S6).

In accordance with the biochemistry depicted in Fig. 1a, each enzyme converted multiple substrates and each substrate was distributed over multiple enzymes (Fig. 2h). In the absence of MCAD, SCAD and LCAD took over the conversion of C6–C10 acyl-CoAs (Fig. 2i). This indicates that the characteristic properties of the mFAO pathway – redundancy of enzymes and competition among substrates for an enzyme – were preserved in the model simulations.

### MCAD is required in the mFAO pathway to protect against substrate overload

**Fig. 3:**
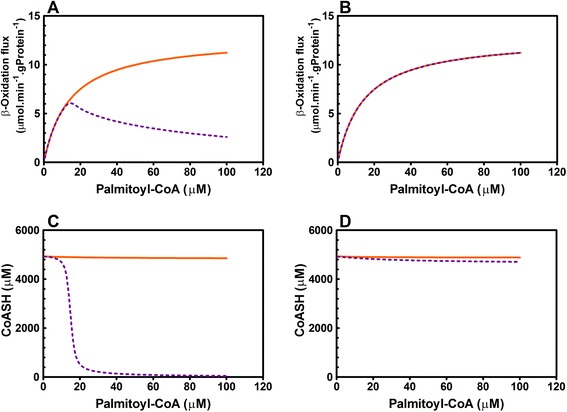
Steady-state rates and CoASH concentrations at increasing concentrations of the substrate palmitoyl-CoA for wild-type (solid orange line) and MCAD-KO (dashed purple line). **a** Steady-state palmitoyl-CoA oxidation rate in the mouse liver model with competition. **b** Steady-state palmitoyl-CoA oxidation rate in the mouse liver model without competition. **c** Steady-state CoASH concentration in the mouse liver model with competition. **d** Steady-state CoASH concentration in the mouse liver model without competition

Next, we studied the effect of fasting on the mFAO pathway in wild-type and MCAD-KO mice by computational modeling. Free fatty acid levels are known to increase upon fasting [22] and we mimicked this by increasing the palmitoyl-CoA concentration. Palmitoyl-CoA is the activated form of the fatty acid palmitate (C16) and it is the first substrate in the computational model. In the wild-type model, the mFAO rate approached a maximum at saturating substrate concentrations (Fig. 3a). When we simulated the MCAD-KO in the model, by setting the *V*_*max*_ value of MCAD to 0, the flux declined steeply when palmitoyl-CoA exceeded 20 μM (Fig. 3a). This coincided with a steep decrease in free coenzyme A (CoASH) (Fig. 3c) due to its sequestration in accumulating CoA esters (Additional file 7: Figure S7A). CoASH is a substrate for the reactions catalyzed by the enzymes carnitine palmitoyltransferase 2 (CPT2), mitochondrial trifunctional protein and MCKAT, and is therefore essential for maintaining mFAO flux. Previously, we have shown that accumulation of CoA esters as such does not necessarily inhibit mFAO flux [16]. It would appear that, when the mFAO pathway became overloaded with substrate, CoA esters accumulated, which caused a steep decline of CoASH and rendered the pathway incapable of maintaining its flux.

We then explored why the flux declined so steeply in the MCAD-KO mouse, while the wild-type was protected against substrate overload. We hypothesized that this was caused by the competition among CoA esters of different chain lengths for a limited set of acyl-CoA dehydrogenases. This competition would become more intense in the MCAD-KO, where the MCAD substrates have to compete with other acyl-CoAs for the remaining enzymes SCAD, LCAD and VLCAD. To test this hypothesis, we adapted the model. In the original model, each reaction was competitively inhibited by substrates and products of different chain lengths (Fig. 1b). We constructed an alternative model version in which each enzyme pool was split into distinct smaller pools, each dedicated to the conversion of a particular chain length and not affected by competing substrates (as in [16]; see Fig. 1b and Methods for a mathematical description; Additional file 8: Model S8). When competition was thus removed, mFAO flux no longer decreased at high palmitoyl-CoA concentrations, nor did we observe the steep decline in CoASH concentration (Fig. 3b, d; Additional file 7: Figure S7B). This proves that, under these conditions in the model, MCAD-KO mitochondria have sufficient catalytic capacity to oxidize fatty acids at the same maximum rate as wild-type mitochondria. In the absence of MCAD, it appeared that, instead, molecular competition – an intrinsic biochemical feature of the pathway topology – was the cause of the pathway’s susceptibility to becoming overloaded and losing its capability to maintain flux at high substrate concentrations.

### The relevance of substrate competition to physiology and clinical outcome

Subsequently, we asked whether substrate competition is of any physiological or clinical relevance. In patients suffering from mFAO deficiencies, serum acylcarnitine profiles are well documented, since they form the basis for diagnosis of the disease and the patient’s metabolic status. They are often considered to be an approximation of mitochondrial acyl-CoA levels, since accumulated CoA esters are converted into carnitine esters by CPT2 (Fig. 1a) and transported into the blood. We therefore studied the acylcarnitine profiles in the competition and non-competition models in more detail.

As in MCAD-deficient patients [20], our MCAD-KO mice accumulated acylcarnitines with chain lengths varying from C6 to C10 (Additional file 2: Figure S2A–C). Both the competition and the non-competition version of the MCAD-deficient model mimicked these elevated levels of medium-chain acylcarnitines, although the patterns differed quantitatively (Additional file 7: Figure S7C, D). These medium-chain acylcarnitines are derived from medium-chain acyl-CoAs, which are the direct substrate of MCAD. Therefore, their accumulation is somewhat trivial and not dependent on competition. We concluded that MCAD deficiency was not the most appropriate condition to distinguish between the two models.

**Fig. 4:**
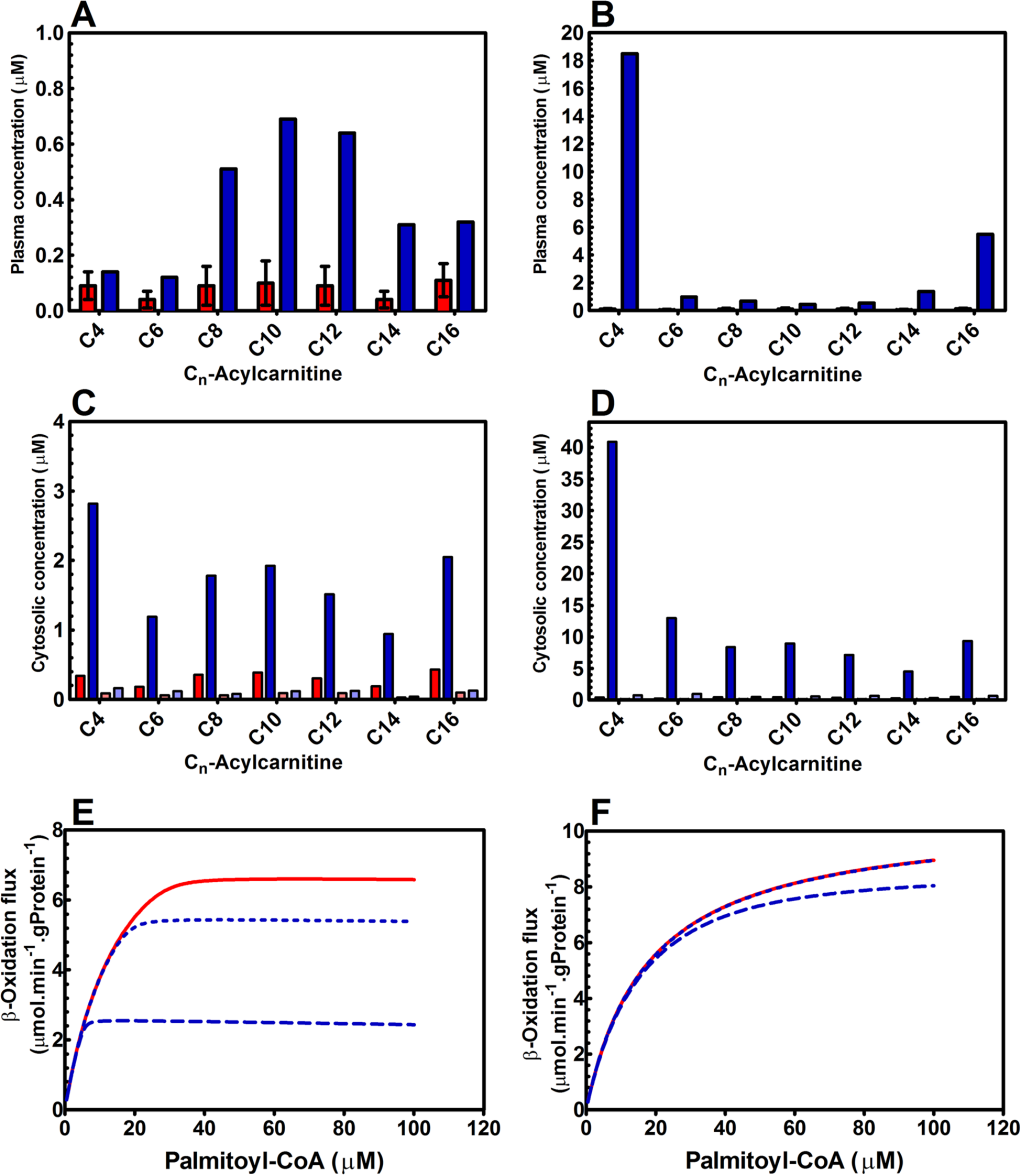
Experimental and simulated acylcarnitine profiles of two MADD patients, differing in disease severity, and the consequences for the simulated mFAO flux in those patients. Panels **a** and **b**: Experimental data for plasma concentrations from a healthy population (red bars; data represents mean ± standard deviation (n = 1750) and the two MADD patients (single time point; blue bars; panel **a**: patient 1; panel **b**: patient 2). Note that the same control data were used for **a** and **b**, but due to the different Y-axis scales, they are barely visible in panel **b**. Panels **c** and **d**: Simulated acylcarnitine profiles for healthy subjects (red bars; FADH_2_ concentration of 0.43 μM), patient 1 (blue bars panel **c**; FADH_2_ concentration 0.6 μM) and patient 2 (blue bars panel **d**; FADH_2_ concentration 0.73 μM). Simulations were performed at a constant sum of FADH_2_ and FAD of 0.77 μM. The dark blue and red bars are the concentrations simulated in the human model with competition and the light blue and red bars are the concentrations simulated in the human model without competition. Panel **e**: Steady-state rates of flux at increasing concentrations of the substrate palmitoyl-CoA for healthy subjects (solid red line; FADH_2_ concentration of 0.43 μM), patient 1 (dotted blue line; FADH_2_ concentration 0.6 μM) and patient 2 (dashed blue line; FADH_2_ concentration 0.73 μM) simulated in the model with competition. Panel **f**: Steady-state rates of flux at increasing concentrations of the substrate palmitoyl-CoA for healthy subjects (solid red line; FADH_2_ concentration of 0.43 μM), patient 1 (dotted blue line; FADH_2_ concentration 0.6 μM) and patient 2 (dashed blue line; FADH_2_ concentration 0.73 μM) simulated in the model without competition

For a more stringent test of the physiological relevance of substrate competition we shifted to MADD. Patients suffering from this disease have a partial deficiency in one of the three electron transfer flavoprotein (ETF) proteins: ETFα, ETFβ or ETF dehydrogenase. These proteins are responsible for the electron transfer between the acyl-CoA dehydrogenases and the mitochondrial electron transport chain [23]. Consequently, the transfer of electrons from the FADH_2_ cofactor of the acyl-CoA dehydrogenases to the electron transport chain (Fig. 1a) is diminished in MADD patients [23]. This leads to a higher FADH_2_ reduction state and inhibits the oxidation of acyl-CoA esters of all chain lengths through simultaneous inhibition of all acyl-CoA dehydrogenases. MADD patients have heterogeneous acylcarnitine profiles. Figure 4a, b shows two such profiles. Compared with healthy controls, both patients had elevated acylcarnitine levels over the entire range of carbon chain lengths. In the first patient (Fig. 4a), levels of medium-chain acylcarnitines were specifically elevated, while in the second (Fig. 4b), butanoylcarnitine (C4) and palmitoylcarnitine (C16) were extremely high.

To simulate the patients’ acylcarnitine profiles, we converted the original rat model into a human model. Since LCAD is not essential in human mFAO [12], it was removed from the model. Kinetic parameters available for human SCAD, MCAD and VLCAD were then incorporated into the model [24, 25]. Subsequently, we estimated a subset of parameters by fitting the model to the average acylcarnitine profile from a healthy population. Since these were plasma values and the model contained cytosolic concentrations of acylcarnitines, the data were converted to cytosolic concentrations as described in the Methods section. The acylcarnitine profiles predicted by the human model (Additional file 9: Model S9) corresponded closely to the acylcarnitine profiles of healthy subjects (Additional file 7: Figure S7E) and CoA esters of various chain lengths were distributed over different enzymes, as expected (Additional file 7: Figure S7F).

First, we simulated the MADD acylcarnitine profiles in the two mFAO models, both including and excluding substrate competition (Additional file 9: Model S9 and Additional file 10: Model S10). To this end, we increased FADH_2_ while keeping the total concentration of FADH_2_ plus FAD constant at 0.77 μM. The patient profiles could be mimicked qualitatively by the model with competition (dark blue bars in Fig. 4c, d). Since the model yields cytosolic liver concentrations, we cannot compare the absolute numbers with levels in patient serum, but we are able to compare the patterns. The different profiles were associated with different FAD reduction states. A profile similar to that of patient 1 – showing accumulation of medium-chain acyl carnitines (Fig. 4a, c) – was calculated at an FADH_2_ concentration of 0.6 μM (78% reduction of total FAD). The FADH_2_ concentration was further increased to 0.73 μM (95% reduction of total FAD) to obtain a profile similar to that of patient 2 – showing characteristic accumulation of C4 and C16 acylcarnitines (Fig. 4b, d). In contrast, in the human model without molecular competition (Additional file 10: Model S10), acylcarnitine concentrations remained low over the whole range of carbon chain lengths, irrespective of the degree of reduction of the FAD pool (light blue bars in Fig. 4c, d). The latter result can be understood intuitively. Accumulation of metabolites occurs when their production and consumption are unbalanced. A diminished capacity of FADH_2_ oxidation affects each cycle of the mFAO pathway to a similar extent and such an imbalance is unexpected. The elevated levels of acylcarnitines over the whole range of chain lengths, as observed in patients, are therefore counterintuitive and require a special mechanism. The fact that the competition model predicted this pattern correctly suggests that this special mechanism might well be substrate competition. This finding also supports the notion that substrate competition is important to explain patient physiology.

Second, we simulated the effect of MADD on mFAO flux and explored how this was influenced by substrate competition. We increased the palmitoyl-CoA concentration gradually at three concentrations of FADH_2_: 0.43 μM (56% reduced) for healthy human liver, 0.6 μM (78% reduced) for patient 1, and 0.73 μM (95% reduced) for patient 2. When the FADH_2_ concentration was increased, the maximum rate through the mFAO pathway decreased (Fig. 4e), with a concomitant reduction in CoASH levels (Additional file 7: Figure S7G). This implies that the mFAO capacity of patient 2 was much more affected than that of patient 1, which is consistent with the patients’ clinical symptoms. Patient 1 had mild clinical symptoms, while patient 2 was severely affected, with congenital anomalies and died 3 days after birth. In contrast, the model without competition predicted only a mild effect of the FAD reduction state on flux, even if the FAD pool was 95% reduced (Fig. 4f). This is incompatible with the patients’ symptoms and further corroborates the importance of substrate competition for patient physiology.

We conclude that substrate competition is the main mechanism that helps to explain the acylcarnitine profiles seen in MADD patients, and that a computational model that includes substrate competition may explain the severity of the disease phenotype in these patients.

## Discussion

In this work, we have shown that deficiencies in the enzymes involved in the mFAO pathway can cause severe loss of pathway robustness. In agreement with our previous work [16], we demonstrated that substrate competition was an essential aspect of the mechanism that caused a steep decline in flux when the pathway was overloaded with substrate. MCAD deficiency aggravated the risk of overload, since the same substrates then competed for fewer isoenzymes. The resulting flux decline was mediated by a self-amplifying depletion of CoASH. We like to emphasize that the current analysis does not yet dissect the sequence of events that explains how metabolite competition leads to a flux decline; this is the subject of our ongoing studies (manuscript in preparation).

In our simulations of MADD patients, substrate competition and CoASH decline resulted in fluxes far below the catalytic capacity that could be realized with the same enzymes in a non-competition model (Fig. 4e, f). CoASH is an essential cofactor, and the maintenance of the CoASH pool is critical for energy metabolism. Chemical inhibition of CoASH biosynthesis in mice is lethal and it has been shown that the primary cause of death in these mice is hypoglycemia [26]. Hence, CoASH provides a direct link between fatty acid metabolism and glucose homeostasis. This suggests that the decline in CoASH due to substrate competition may also be the underlying cause of the low blood glucose levels seen in MCAD-deficient patients during a metabolic crisis.

Mitchell et al. [27] reviewed the widespread relevance of *C*o*A s*equestration, *to*xicity and *r*edistribution (CASTOR) in inborn enzyme deficiencies, not only in mFAO, but also, for instance, in branched-chain amino-acid metabolism. According to these authors, a common denominator of these diseases is the accumulation of CoA esters and the associated depletion of CoASH, which leads to the risk of life-threatening hypoglycemia. They proposed that alterations in CoA metabolism might displace the patient’s “set point” such that minor physiological fluctuations, such as a surplus of CoA-dependent substrates, might induce a metabolic crisis. Although very plausible, since their publication in 2008, these ideas have hardly been followed up. This may be because CoASH and CoA-ester concentrations in the liver and other patient tissues are relatively inaccessible. Here, we have shown how computational modeling can fill this gap. The main progress of our work beyond the CASTOR hypothesis is that that we provide a molecular mechanism in which substrate competition among acyl-CoAs for common enzymes strongly amplifies the depletion of CoASH: levels of CoASH did not decline gradually as a function of palmitoyl-CoA substrate, but declined steeply beyond a threshold value of substrate (Fig. 3c). In this respect, it should be noted that not only dietary nutrients, but also xenobiotics, drugs, and intestinal fermentation products, such as butyrate and propionate, depend on CoA for their metabolism [28, 29]. For a complete view of CoA homeostasis it will be necessary to integrate the metabolism of all these substances into the model.

The clinical spectrum of patients with a deficiency in mFAO varies considerably, ranging from neonatal death to patients who remain asymptomatic throughout life. Even a group of patients who all have the same MCAD mutation and who have no residual MCAD activity have been shown to exhibit this full range of clinical presentations [30]. In part, this may be due to compensatory mechanisms, such as increased expression of SCAD or peroxisomal β-oxidation enzymes, which can take over the catalytic function of MCAD. In the MCAD-KO mice we did not observe adaptations in SCAD, however. Alternatively, other CoA-dependent processes may be downregulated, or CoA-liberating enzymes, such as acyl-CoA esterases [31–33], upregulated. For the broader group of mFAO disorders it is likely that the clinical heterogeneity may also be attributed to differences in the residual activity of the defective protein. However, for the other example considered in the current study (MADD), the residual enzyme activity is not routinely measured in patients, since the ETF and ETF dehydrogenase assays require anaerobic conditions [34]. Finally, the different clinical presentations may be due to different exposures to CoA-dependent nutrients, intestinal fermentation products, or xenobiotics.

Clearly, the disease severity and optimal treatment, and possibly also the susceptibility to nutritional or xenobiotic factors, differ between individual patients and depend not only on the primary disease-related mutation, making mFAO disorders attractive for a personalized systems medicine approach. Because liver and muscle tissues are not readily accessible for diagnostic purposes, functional tests, such as tests for enzyme activities, mFAO rates, or compensatory gene expression, must be performed on less relevant cell types such as fibroblasts. Instead, we do have access to serum metabolic profiles, and metabolomics methodology is developing rapidly [35]. Making the link between these “circumstantial” data and the disease mechanism in the affected tissues requires computational modeling [36]. Recently, personalized genome-scale stoichiometric metabolic models have been developed to identify patient-specific drug targets [37, 38]. A limitation of these models, however, is that they lack kinetics, while more complex disease mechanisms, such as the substrate competition mechanism highlighted here, do depend on complex enzyme kinetics.

To our knowledge, this is one of the first studies in which a dynamic computational model based on detailed biochemistry has been used to describe individual patients and differentiate between them (see [39] for another example). It will be important to validate the approach in a larger cohort of patients. If this is successful, the model will enable us to assess the potential of treatment options previously considered, such as the administration of carnitine or CoA precursors [40], or to suggest entirely novel treatments such as medications that interfere in redox metabolism [16]. To obtain a fully-fledged patient-specific risk and treatment profile, it will be necessary to expand the approach to include surrounding metabolic pathways such as citric acid cycle, oxidative phosphorylation [39, 41], peroxisomal fatty-acid oxidation, and CoA-dependent pathways. Nevertheless, it is a promising first step towards a patient-specific risk assessment for a group of severe metabolic disorders. For now, the computational model has allowed us to gain insight into a disease mechanism that is normally considered inaccessible due to limited tissue sampling and the absence of routine assays for CoA esters [27].

## Conclusion

Systems medicine holds great promise for understanding the mechanisms of complex diseases and improving treatments tailored to individual patients. Here, we used computer modeling to analyze metabolic data from knockout mice and patients with mFAO disorders, and explain why patient physiology is not robust against metabolic challenges. Enzymes in the mFAO accept multiple substrates of different chain lengths. This leads to molecular competition among the substrates. We demonstrated that substrate competition aggravates metabolic impairment in these disorders more than should be expected from the defect alone. Our detailed biochemical model can explain the characteristic metabolite profiles seen in these patients and predict the severity of their symptoms. To our knowledge, this is one of the first descriptions of a detailed dynamic model of metabolism being applied to individual patient data. Our results therefore provide a proof of principle for the systems medicine approach.

## Methods

### Animal experiments

MCAD-KO mice on the mixed 129P2xC57BL6/J background [17] strain were crossed for five generations with wild-type C57BL6/J mice. Heterozygous breeding pairs were taken from the fifth generation offspring to generate the MCAD-KO and wild-type littermates used in this study.

Male mice were used for experiments at the age of 2–4 months. Mice were fed commercially available laboratory chow (ABDiets, Woerden, The Netherlands). For experiments under fasting conditions, mice were placed in a clean cage at 9 pm and terminated and dissected 12 hours later by cardiac puncture under isoflurane anesthesia. On the day of sacrifice, bloodspots were obtained by cardiac puncture for acylcarnitine analysis. A small piece of liver tissue was collected and snap-frozen in liquid nitrogen for mRNA analysis. Part of the liver was freeze-clamped and stored at –80 °C. The rest of the liver tissue was collected in a buffer containing 250 mM sucrose and 10 mM Tris (pH 7.0) and used for the isolation of mitochondria.

### Dynamic flux and metabolite profiles in isolated mitochondria

Mitochondria were isolated from liver tissue of male C57Bl/6 J wild-type and MCAD-KO mice (2–4 months old) according to the method of Mildaziene et al. [42]. The oxygen consumption rate of uncoupled mitochondria was measured by incubating them with substrate (either palmitoylcarnitine (C16) plus malate, or octanoylcarnitine (C8) plus malate) in the presence of ADP, l-carnitine and FCCP at 37 °C in a stirred, two-channel high-resolution Oroboros oxygraph-2 k (Oroboros, Innsbruck, Austria). See Van Eunen et al. [16] for an elaborate description of the procedure. Samples to measure the acylcarnitine were quenched in acetonitrile (10 μL sample in 100 μL acetonitrile).

### Mass spectrometry analysis of acylcarnitines

Acylcarnitine concentrations were measured in liver homogenates and mitochondrial samples according to Derks et al. [20].

### Quantitative PCR

Total RNA was isolated from homogenates of snap-frozen liver tissue of adult male MCAD-KO (n = 4) and wild-type C57BL6/J (n = 4) mice with Tri reagent (according to the manufacturer’s instructions; Sigma-Aldrich, St Louis, MO). Total mRNA was quantified on a NanoDrop ND-100 UV-Vis spectrophotometer (NanoDrop Technologies, Wilmington, DE, USA). cDNA was synthesized by a reverse transcription procedure according to the manufacturer’s protocols (Sigma-Aldrich). cDNA was amplified with the primers and probes listed in Additional file 11: Table S11. Real-time PCR was performed on an ABI-Prism 7700 fast PCR system (Applied Biosystems, Foster City, CA, USA). Transcript levels were calculated relative to the expression of the housekeeping gene 36B4, and normalized for expression levels of wild-type mice.

### Targeted proteomics of mFAO proteins

Absolute quantification of mFAO proteins was done by targeted proteomics [43]. Isotopically labeled standards were ordered from Polyquant in the form of purified, synthetic proteins consisting of peptide concatamers with ^13^C-labeled lysine and arginine, derived from the proteins of interest. The protein concatamers (QconCATs) were designed to quantify each of the proteins with two or three standard peptides. See for a detailed description of the method [44]. The selected peptides are listed in Additional file 12: Table S12.

### Enzyme activity

Enzyme activity assays were done in either liver homogenate or mitochondrial extracts. The reported enzyme activities represent the summed activity of all isoenzymes in the extract at saturating substrate concentrations and are expressed per liver or mitochondrial protein (μmoles of substrate converted per minute per milligram extracted protein). Tissues or mitochondria were disrupted by sonication (30 one-second pulses, 10–13 Watt with one-second breaks; samples were on ice during the entire procedure) in either phosphate-buffered saline (137 mM NaCl, 2.7 mM KCl, 10 mM Na_2_HPO_4_, 1.8 mM KH_2_PO_4_) or in a buffer with 250 mM sucrose, 10 mM Tris pH 7.0, and 1 mM DTT. All enzyme activities, except for that of MCAD, were measured in freshly prepared extracts at 37 °C in a Synergy H4 plate reader (BioTek, Winooski, US). Three or four different dilutions in 10 mM phosphate buffer (pH 7.0) with DTT were analyzed to check for linearity. The acyl-CoA dehydrogenase activity was so low that only the undiluted sample could be measured. For the remaining enzymes we took the average of 2 or 3 dilutions in the linear area. Protein determinations were carried out with the bicinchoninic acid kit (BCA Protein Assay Kit; Pierce, Thermo Fisher Scientific, Rockford, IL, USA) with BSA (2 mg/mL stock solution; Pierce) as a standard; 1 mM DDT was added to the standard if it was also present in the extract. For all assays the reaction mixtures without start reagent were pre-warmed at 37 °C.

#### 3-Phenylpropionyl-CoA dependent MCAD

MCAD enzyme activity was measured at 37 °C in medium containing 100 mM phosphate buffer (pH 8.0), 1 mM ferrocenium hexafluorophosphate and 0.4 mM 3-phenylpropionyl CoA (start reagent). Reactions were terminated after 20 minutes. The product cinnamoyl-CoA was measured on an HPLC system with UV detection (Waters, Milford, MA, USA).

#### Acyl-CoA dehydrogenase (ACAD) (modified from [45])

The reaction mixture contained 100 mM potassium phosphate buffer (pH 7.4), 0.4 mM ferricenium hexafluorophosphate, 0.5 mM N-ethylmaleimide, 0.1 mM EDTA, 0.1% Triton x-100, and 0.5 mM C_n_Acyl-CoA (start reagent; where n denotes number of carbon atoms of the unbranched acyl chain). The reduction of ferricenium hexafluorophosphate was followed over time by measuring the absorbance at 300 nm.

#### Medium/short-chain hydroxyacyl-CoA dehydrogenase (M/SCHAD) (modified from [46])

The reaction mixture contained 0.1 M Tris-HCl (pH 10.0), 1 mM NAD^+^, and 0.1 mM hydroxybutyryl-CoA (start reagent). The production of NADH was monitored over time at 340 nm.

#### Crotonase (CROT)

The assay mixture contained 0.1 M Tris-HCl (pH 10.0), 1 mM NAD^+^, and 0.15 mM Crotonyl CoA (start reagent). The production of NADH was monitored over time at 340 nm. This assay records the combined activity of CROT and M/SCHAD and was only used to check whether CROT activity was present in the samples. The measured activity was not used in the model.

#### Medium-chain ketoacyl-CoA thiolase (MCKAT) (modified from [47])

The assay mixture contained 0.1 M Tris-HCl (pH 8.0), 25 mM MgCl_2_, 50 mM KCl, 0.05 mM acetoacetyl CoA and 0.05 mM CoA (start reagent). Measuring the absorbance at 303 nm over time determined the consumption rate of acetoacetyl CoA.

### Computational methods

The computational model described in Van Eunen et al. [16] was converted to sbml format and imported into COPASI software version 4.11.65 [48] for parameter estimation as well as for steady-state and time simulations. The concentrations in the model are in μM and the time unit is minutes. Fluxes of individual enzymes are in μmol.min^–1^.mg mitochondrial protein^–1^. The rate through the NADH oxidation reaction (vnadhsink) is taken as the rate through the mFAO pathway.

For the mouse model the *V*_*max*_ values obtained from the enzyme activity measurements were used as input for the model. A subset of kinetic constants (Additional file 4: Table S4) was fitted to the combined dataset of acylcarnitine time courses in isolated mitochondria of wild-type and MCAD-KO mouse livers. The data of all four experimental conditions, i.e., wild-type and MCAD-KO with either palmitoyl- or octanoylcarnitine as the substrate, were used in one fitting round to obtain a single set of parameters. The subset of parameters was defined based on the sensitivity of the acylcarnitine concentrations to the specific parameters. From this list of parameters we selected the parameters that were identifiable. This means that we included only the parameters that gave a value with a small standard error when they were fitted to the data. Parameter values to be fitted were allowed to vary between the lower and upper boundary as indicated in Additional file 4: Table S4. For parameters that were deconvolved from the measured enzyme kinetics, the boundaries were set to 20% higher or lower than the measured value. The specificity factors always had an upper boundary of 1, since it indicates the maximum activity for that specific chain-length. The boundaries of the remaining parameters were set such that the characteristic properties of the mFAO pathway – redundancy of enzymes and competition among substrates for an enzyme – were preserved in the model simulations. Initial concentrations of the acylcarnitines were allowed to vary between the measured value ± 20% and the *V*_*max*_ value of MCAD in the wild-type condition between the measured value ± 10%.

To humanize the model, LCAD was removed and parameters for which the human values were known were incorporated (Additional file 13: Table S13). A subset of kinetic constants (Additional file 14: Table S14) was fitted to the average acylcarnitine profile from a healthy population. Since these were plasma values and the model contained cytosolic concentrations of acylcarnitines, the concentrations were converted from plasma to cytosolic concentrations. This was done by keeping the steady-state palmitoylcarnitine (C16) concentration of the model and adapting the concentrations of the other chain lengths so that they had the same ratio as that seen in the plasma data. The subset of parameters to be fitted was defined based on the sensitivity of the acylcarnitine concentrations to the specific parameters. From this list of parameters we selected the parameters that were identifiable. This means that we included only the parameters that gave a value with a small standard error when they were fitted to the data. Parameter values to be fitted were allowed to vary between the lower and upper boundary as indicated in the Additional file 14: Table S14. The boundaries of the remaining parameters were set such that the characteristic properties of the mFAO pathway – redundancy of enzymes and competition among substrates for an enzyme – were preserved in the model simulations.

Steady state in both the mouse and human model was calculated with a fixed palmitoyl-CoA concentration of 25 μM, which was the substrate of the model, unless indicated otherwise. The concentrations of the boundary metabolites were also fixed and are given in Additional file 15: Table S15. For both the mouse and human model, the obtained steady-state concentrations of the acylcarnitines and CoASH as well as the flux through the mFAO (expressed as flux to NADH) are presented in Additional file 15: Table S15.

Parameter estimation was done in COPASI [48] by applying the Levenberg-Marquardt algorithm (iteration limit 200, tolerance 1 × 10^–6^). The extracellular concentrations of the acylcarnitines were compared with the experimental data and the least sum of squares was the objective function to be minimized:

1 $$ E(P)={\displaystyle \sum_{i,j}}{\omega}_j\cdot {\left({x}_{i,j}-{y}_{i,j}(P)\right)}^2 $$

in which *E* is the objective value, *P* is the tested parameter set, *x*_*i,j*_ is a point in the dataset, and *y*_*i,j*_(*P*) is the corresponding simulated value. The indices *i* and *j* represent time and metabolite species in the dataset. The weight factor for each data column is given by *ω*_*j*_ and was set to a fixed value of 1. Additional file 4: Table S4, Additional file 14: Table S14, and Additional file 15: Table S15 list (2) the boundary metabolite concentrations used for steady-state calculations, (2) the fitted parameters and the imposed constraints, (3) the steady-state fluxes, and (4) metabolite concentrations for both the mouse and human model.

The competition was removed from the model such that a percentage of each enzyme was dedicated to a particular chain length and that the competitive inhibition term was removed from the equation. Figure 1b gives an example of an equation with and without competition. Enzymes were partitioned such that at 25 μM of palmitoyl CoA the flux distribution among parallel enzymes was similar in the models with and without competition. This was different for the wild-type and MCAD-KO model, since the flux is redistributed over the remaining acyl-CoA dehydrogenases when MCAD is lost. Additional file 16: Table S16 contains the partition factors for both the mouse and human model.

### Statistical analysis

Differences between normally distributed continuous data were analyzed using parametric tests, and data that were not normally distributed were analyzed using non-parametric tests. The significance level was set at *P* < 0.05. Statistical analyses were performed using GraphPad Prism software (GraphPad Software Inc., version 5.00, 2007).

## Additional files

Additional file 1: Table S1. Mouse characteristics after 12 hours of fasting. (PDF 67 kb)
Additional file 2: Figure S2. Comparison of the characteristics of wild-type mice and MCAD-KO mice. (PDF 179 kb)
Additional file 3: Text S3. Obtaining the chain-length specificity factors and *V*_*max*_ values for VLCAD, LCAD, MCAD, and SCAD. (PDF 254 kb)
Additional file 4: Table S4. Estimated parameters for the mouse model. (PDF 103 kb)
Additional file 5: Model S5. Mouse-liver fatty acid beta-oxidation competition. (XML 470 kb)
Additional file 6: Table S6. Flux control coefficients of the maximum flux over the timescale of the experiment (25 minutes). (PDF 192 kb)
Additional file 7: Figure S7. Steady-state simulation data of the mouse and human liver mFAO. (PDF 243 kb)
Additional file 8: Model S8. Mouse-liver fatty acid beta-oxidation no competition. (XML 225 kb)
Additional file 9: Model S9. Human-liver fatty acid beta-oxidation competition. (XML 536 kb)
Additional file 10: Model S10. Human-liver fatty acid beta-oxidation no competition. (XML 233 kb)
Additional file 11: Table S11. List of primers used for the real-time PCR. (PDF 555 kb)
Additional file 12: Table S12. List of peptides used for targeted proteomics of mFAO proteins. (PDF 146 kb)
Additional file 13: Table S13. Parameters for which we incorporated a human value to humanize the rat model. (PDF 87 kb)
Additional file 14: Table S14. Estimated parameters for the human model. (PDF 102 kb)
Additional file 15: Table S15. Steady-state characteristics of the computer models. (PDF 93 kb)
Additional file 16: Table S16. Partition factor of the mouse and human model of mFAO. (PDF 100 kb)

## Abbreviations

ACAD: acyl-CoA dehydrogenase (in the context of this paper referring to SCAD, MCAD, LCAD, and VLCAD together)
CPT2: carnitine palmitoyltransferase 2
CROT: crotonase
ETF: electron transfer flavoprotein
KO: knockout
LCAD: long-chain acyl-CoA dehydrogenase
M/SCHAD: medium/short-chain hydroxyacyl-CoA dehydrogenase
MADD: multiple acyl-CoA dehydrogenase
MCAD: medium-chain acyl-CoA dehydrogenase
MCKAT: medium-chain ketoacyl-CoA thiolase
mFAO: mitochondrial fatty-acid oxidation
SCAD: short-chain acyl-CoA dehydrogenase
VLCAD: very-long-chain acyl-CoA dehydrogenase
