# Supplementary material for: Living on the edge: substrate competition explains loss of robustness in mitochondrial fatty-acid oxidation disorders
Source: BMC Biol. 2016 Dec 7;14:107. doi: 10.1186/s12915-016-0327-5 (PMC5142382; doi:10.1186/s12915-016-0327-5)
Supplement: Additional file 1: Table S1. — Mouse characteristics after 12 hours of fasting. (PDF 67 kb) [file 12915_2016_327_MOESM1_ESM.pdf]

Supplemental Table S1

Mouse characteristics after 12 hours of fasting

Median and range are presented. ‡ Blood samples of two mice were pooled for this analysis. Three pooled plasma samples were analyzed per genotype. ** $p < 0.01$.

	**Wild type**	**MCAD-KO**
Body weight (g)	23.9<br>(17.8-27.1)	23.0<br>(20.7-27.1)
Body weight loss upon fasting (%)	12.4<br>(7.8-19.9)	11.2<br>(10.0-13.8)
Body weight/liver ratio	0.047<br>(0.043-0.052)	0.050<br>(0.041-0.053)
Glucose (mmol/l)	5.8<br>(4.6-7.8)	5.3<br>(4.6-6.8)
β-OH-butyrate (mmol/l)‡	2.30<br>(1.59 – 2.60)	1.35<br>(1.29 – 1.99)
Free fatty acids (mmol/l) ‡	432<br>(410 – 714)	583<br>(516 – 598)
C6-carnitine bloodspot (µmol/l)	0.06<br>(0.04 – 0.07)	0.14<br>(0.09 – 0.30)**
C8-carnitine bloodspot (µmol /l)	0.07<br>(0.06 – 0.42)	0.49<br>(0.46 – 0.56)**
C10-carnitine bloodspot (µmol /l) ‡	0.03<br>(0.02 – 0.06)	0.07<br>(0.05 – 0.09)**
C10:1-carnitine bloodspot (µmol /l) ‡	0.02<br>(0.01 – 0.02)	0.22<br>(0.10 – 0.26)**
C8/C10 ratio bloodspot	3.2<br>(2.4 – 6.9)	7.6<br>(5.3 – 9.3)**
