# Supplementary material for: Living on the edge: substrate competition explains loss of robustness in mitochondrial fatty-acid oxidation disorders
Source: BMC Biol. 2016 Dec 7;14:107. doi: 10.1186/s12915-016-0327-5 (PMC5142382; doi:10.1186/s12915-016-0327-5)

Supplemental Figure S2

Comparison of the characteristics of wild-type mice (orange bars) and MCAD-KO mice (purple bars).

A: The ratio of C8/C10 acylcarnitines in blood and liver mice after 12 h fasting. Data represents median, the box extends from the 25th to 75th percentile and the whiskers extends from the minimum to the maximum value (n = 6). Concentrations of medium-chain acylcarnitines in blood (B; data represents median, the box extends from the 25th to 75th percentile and the whiskers extends from the minimum to the maximum value; n = 6) and liver homogenate (C; data represents median, the box extends from the 25th to 75th percentile and the whiskers extends from the minimum to the maximum value; n = 6) of mice after 12 h fasting. D: MCAD activity for the MCAD-specific substrate PP-CoA per mg liver protein. Data represents median and individual data points (n = 3). E: Oxidation rate of palmitoylcarnitine (C16) and octanoylcarnitine (C8) in isolated mitochondria of fed mice. Data represents median, the box extends from the 25th to 75th percentile and the whiskers extends from the minimum to the maximum value (n = 7). F: Relative changes in the transcript levels of the genes involved in mFAO. Data represents median and individual data points (n = 4). G: Total activity of mFAO enzymes for their substrate with four carbon atoms (C4) per mg mitochondrial protein measured in extracts from mitochondria isolated from mouse liver. Data represents median and individual data points (n = 4). ACAD: acyl-CoA dehydrogenase; CROT: crotonase; M/SCHAD: medium/short-chain hydroxyacyl-CoA dehydrogenase; MCKAT: medium-chain ketoacyl-CoA thiolase.

* $p < 0.05$, ** $p < 0.01$.

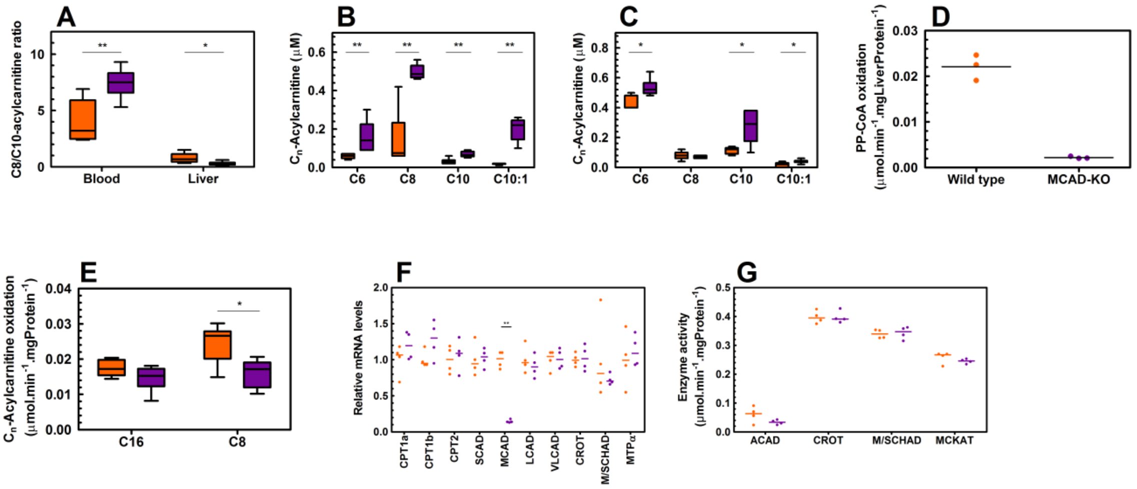

Supplement: Additional file 2: Figure S2. — Comparison of the characteristics of wild-type mice and MCAD-KO mice. (PDF 179 kb) [file 12915_2016_327_MOESM2_ESM.pdf]
