# Supplementary material for: Living on the edge: substrate competition explains loss of robustness in mitochondrial fatty-acid oxidation disorders
Source: BMC Biol. 2016 Dec 7;14:107. doi: 10.1186/s12915-016-0327-5 (PMC5142382; doi:10.1186/s12915-016-0327-5)
Supplement: Additional file 3: Text S3. — Obtaining the chain-length specificity factors and V max values for VLCAD, LCAD, MCAD, and SCAD. (PDF 254 kb) [file 12915_2016_327_MOESM3_ESM.pdf]

Supplemental Text S3

Obtaining the chain-length specificity factors and V_{max} values for VLCAD, LCAD, MCAD and SCAD

We deconvoluted the total acyl-CoA dehydrogenases activities for the various chain lengths to obtain the specificity for the chain length as well as the V_{max} values for SCAD, MCAD, LCAD and VLCAD. In these calculations, we set MCAD to zero in the MCAD-KO dataset and assumed that the activities of the other ACADs were identical in MCAD-KO and wild type since no significant differences were observed in the proteomics data. The set of equations (see below) was solved in Excel, which uses the Generalized Reduced Gradient Nonlinear Solver algorithm (Lasdon et al., 1978). The table below shows the parameter values that were incorporated in the model.

$$Vwt_{ACAD,C4} = Sf_{SCAD,C4} \cdot V_{SCAD} + Sf_{MCAD,C4} \cdot V_{MCAD} \quad [1]$$

$$Vwt_{ACAD,C6} = Sf_{SCAD,C6} \cdot V_{SCAD} + Sf_{MCAD,C6} \cdot V_{MCAD} \quad [2]$$

$$Vwt_{ACAD,C8} = Sf_{MCAD,C8} \cdot V_{MCAD} + Sf_{LCAD,C8} \cdot V_{LCAD} \quad [3]$$

$$Vwt_{ACAD,C10} = Sf_{MCAD,C10} \cdot V_{MCAD} + Sf_{LCAD,C10} \cdot V_{LCAD} \quad [4]$$

$$Vwt_{ACAD,C12} = Sf_{MCAD,C12} \cdot V_{MCAD} + Sf_{LCAD,C12} \cdot V_{LCAD} + Sf_{VLCAD,C12} \cdot V_{VLCAD} \quad [5]$$

$$Vwt_{ACAD,C14} = Sf_{LCAD,C14} \cdot V_{LCAD} + Sf_{VLCAD,C14} \cdot V_{VLCAD} \quad [6]$$

$$Vwt_{ACAD,C16} = Sf_{LCAD,C16} \cdot V_{LCAD} + Sf_{VLCAD,C16} \cdot V_{VLCAD} \quad [7]$$

$$Vmcad\text{-}ko_{ACAD,C4} = Sf_{SCAD,C4} \cdot V_{SCAD} \quad [8]$$

$$Vmcad\text{-}ko_{ACAD,C6} = Sf_{SCAD,C6} \cdot V_{SCAD} \quad [9]$$

$$Vmcad\text{-}ko_{ACAD,C8} = Sf_{LCAD,C8} \cdot V_{LCAD} \quad [10]$$

$$Vmcad\text{-}ko_{ACAD,C10} = Sf_{LCAD,C10} \cdot V_{LCAD} \quad [11]$$

$$Vmcad\text{-}ko_{ACAD,C12} = Sf_{LCAD,C12} \cdot V_{LCAD} + Sf_{VLCAD,C12} \cdot V_{VLCAD} \quad [12]$$

$$Vmcad\text{-}ko_{ACAD,C14} = Sf_{LCAD,C14} \cdot V_{LCAD} + Sf_{VLCAD,C14} \cdot V_{VLCAD} \quad [13]$$

$$Vmcad\text{-}ko_{ACAD,C16} = Sf_{LCAD,C16} \cdot V_{LCAD} + Sf_{VLCAD,C16} \cdot V_{VLCAD} \quad [14]$$

Measured parameter values and parameter values obtained after deconvolution of the total acyl-CoA dehydrogenases activities for the various chain lengths.

Parameter	Obtained value
V_{MSCHAD}[a]	0.34
V_{MCKAT}[a]	0.25
V_{SCAD}[b]	0.036
V_{MCAD}[b]	0.027
V_{LCAD}[b]	0.047
V_{VLCAD}[b]	0.007
$Sf_{SCAD,C4}$[b,c]	0.936
$Sf_{SCAD,C6}$[b,c]	1.000
$Sf_{MCAD,C4}$[b,c]	0.934
$Sf_{MCAD,C6}$[b,c]	0.609
$Sf_{MCAD,C8}$[b,c]	0.742
$Sf_{MCAD,C10}$[b,c]	1.000
$Sf_{MCAD,C12}$[b,c]	0.133
$Sf_{LCAD,C8}$[b,c]	0.689
$Sf_{LCAD,C10}$[b,c]	0.741
$Sf_{LCAD,C12}$[b,c]	1.000
$Sf_{LCAD,C14}$[b,c]	0.862
$Sf_{LCAD,C16}$[b,c]	0.844
$Sf_{VLCAD,C12}$[b,c]	0.106
$Sf_{VLCAD,C14}$[b,c]	0.572
$Sf_{VLCAD,C16}$[b,c]	1.000

[a] Measured parameter values

[b] Deconvoluted parameter values

[c] Parameters that were included in the parameter estimation procedure
