# Supplementary material for: Living on the edge: substrate competition explains loss of robustness in mitochondrial fatty-acid oxidation disorders
Source: BMC Biol. 2016 Dec 7;14:107. doi: 10.1186/s12915-016-0327-5 (PMC5142382; doi:10.1186/s12915-016-0327-5)
Supplement: Additional file 4: Table S4. — Estimated parameters for the mouse model. (PDF 103 kb) [file 12915_2016_327_MOESM4_ESM.pdf]

Supplemental Table S4

Estimated parameters for the mouse model.

List of the parameters with lower and upper boundaries, which were estimated when the model was fitted to the experimental data. The set of parameters to be fitted was chosen based on a sensitivity analysis. The set contains the parameters for which the acylcarnitine concentrations were most sensitive.

Parameter	Original value	Lower boundary	Upper boundary	Estimated value
sfcpt2C16	0.85	1.00E-6	1.00	1.00
sfcpt2C14	1.00	1.00E-6	1.00	1.00
sfcpt2C12	0.95	1.00E-6	1.00	0.42
sfcpt2C10	0.95	1.00E-6	1.00	0.39
sfcpt2C8	0.35	1.00E-6	1.00	0.20
sfcpt2C6	0.15	1.00E-6	1.00	0.02
sfcpt2C4	0.01	1.00E-6	1.00	0.002
Kmcpt2C16AcylCoAMAT	38.0	1.00E-6	1.00E+6	2.75
Kmcpt2C14AcylCoAMAT	38.0	1.00E-6	1.00E+6	19.1
Kmcpt2C12AcylCoAMAT	38.0	1.00E-6	1.00E+6	15.0
Kmcpt2C10AcylCoAMAT	38.0	1.00E-6	1.00E+6	326
Kmcpt2C8AcylCoAMAT	38.0	1.00E-6	1.00E+6	140235
Kmcpt2C6AcylCoAMAT	38.0	1.00E-6	1.00E+6	79.4
Kmcpt2C4AcylCoAMAT	38.0	1.00E-6	1.00E+6	28116
KmvlcadC16AcylCoAMAT	6.50	1.00	6.5	6.50
KmvlcadC14AcylCoAMAT	4.00	1.00	8	8.00
KmvlcadC12AcylCoAMAT	2.70	1.00	100	1.00
KmlcadC16AcylCoAMAT	2.50	1.00	100	2.52
KmlcadC14AcylCoAMAT	7.40	1.00	100	46.3
KmlcadC12AcylCoAMAT	9.00	1.00	100	35.3
KmlcadC10AcylCoAMAT	24.3	1.00	100	23.4
KmmcadC12AcylCoAMAT	5.70	1.00	100	63.6
KmmcadC10AcylCoAMAT	5.40	1.00	100	1.00
KmmcadC8AcylCoAMAT	4.00	1.00	100	1.00
KmmcadC6AcylCoAMAT	9.40	1.00	100	4.10
KmscadC4AcylCoAMAT	10.7	1.00	100	5.78
sfvlcadC16	1.00	0.80	1.00	0.80
sfvlcadC14	0.57	0.46	0.69	0.46
sfvlcadC12	0.11	0.08	0.13	0.08
sflcadC16	0.84	0.68	1.00	0.68
sflcadC14	0.86	0.69	1.00	0.69
sflcadC12	1.00	0.80	1.00	0.80
sflcadC10	0.74	0.59	0.89	0.59
sflcadC8	0.69	0.55	0.83	0.72
sfmcadC12	0.13	0.11	0.16	0.15
sfmcadC10	1.00	0.80	1.00	1.00
sfmcadC8	0.74	0.59	0.89	0.89
sfmcadC6	0.61	0.49	0.73	0.73
sfmcadC4	0.94	0.75	1.00	1.00
sfscadC6	1.00	0.80	1.00	1.00
sfscadC4	0.94	0.75	1.00	1.00
KmmckatC6KetoacylCoAMAT	6.7	0.67	67	0.67
KmmckatC4AcetoacylCoAMAT	12.4	1.24	124	1.24
