# Supplementary material for: Living on the edge: substrate competition explains loss of robustness in mitochondrial fatty-acid oxidation disorders
Source: BMC Biol. 2016 Dec 7;14:107. doi: 10.1186/s12915-016-0327-5 (PMC5142382; doi:10.1186/s12915-016-0327-5)
Supplement: Additional file 6: Table S6. — Flux control coefficients of the maximum flux over the timescale of the experiment (25 minutes). (PDF 192 kb) [file 12915_2016_327_MOESM6_ESM.pdf]

Supplemental Table S6

Flux control coefficients of the maximum flux over the timescale of the experiment (25 minutes)

Non-stationary flux control coefficients (FCC) were calculated for the time point when the NADH production flux (vnadhsink) reached maximum value over the a time course of 25 minutes. A time course instead of a steady state was chosen to mimic the experimental analysis, in which the system does not reach a steady state (Figure 2C-G). The flux control coefficient $C_{v_i}^{J} = \frac{d \ln J}{d \ln v_i}$ was approximated by increasing the V_{max} of a specific reaction i by 0.1% and calculating the initial effect on the reaction rate v_i as well as on the flux J at the same time point as in the reference condition. Since we calculated the flux control coefficients in a dynamic condition the sum of the flux control coefficients does not equal 1. The enzymes with the highest control were indicated in bold.

**Enzyme/Reaction**	**C16-carnitine as substrate**<br>FCC 0.1% increase	**C8-carnitine as substrate**<br>FCC 0.1% increase
CACT	0.00	-0.21
CPT2	0.03	-0.11
VLCAD	0.04	-0.25
LCAD	**0.42**	-0.20
MCAD	0.11	**0.23**
SCAD	0.00	-0.22
CROT	-0.01	-0.22
M/SCHAD	0.00	-0.25
MCKAT	0.07	-0.17
MTP	0.19	-0.12
FADHSINK	0.00	-0.25
NADHSINK	0.00	-0.26
ACECOASINK	0.00	-0.25
