# Supplementary material for: Living on the edge: substrate competition explains loss of robustness in mitochondrial fatty-acid oxidation disorders
Source: BMC Biol. 2016 Dec 7;14:107. doi: 10.1186/s12915-016-0327-5 (PMC5142382; doi:10.1186/s12915-016-0327-5)

Supplemental Figure S7

Steady-state simulation data of the mouse and human liver mFAO

A-B: Steady-state total CoA-ester concentrations at increasing concentrations of the substrate palmitoyl-CoA in mouse liver mFAO. Panel A: Steady-state total CoA-ester concentrations in the mouse liver model with competition, for wild type (solid orange line) and MCAD-KO (dashed purple line). Panel B: Steady-state total CoA-ester concentrations in the mouse-liver model without competition, for wild type (solid orange line) and MCAD-KO (dashed purple line).

C-D: Steady-state acylcarnitine profile for MCAD-KO (purple bars) versus wild-type (orange bars) mice at a palmitoyl-CoA concentration of 10 μM, in the model with competition (panel C) and without competition (panel D). The mFAO rate in both wild type and MCAD-KO is similar at this concentration of palmitoyl-CoA.

E: Acylcarnitine concentrations of the humanized model (before parameter estimation; green bars), the experimental data of a healthy population (turquoise bars) and after parameter estimation to fit the experimental data (red bars).

F: Distribution of the steady-state flux across the various chain lengths for the mFAO enzymes simulated in the computational model of human liver mFAO after parameter estimation.

G: Steady-state CoASH concentrations at increasing concentrations of the substrate palmitoyl-CoA in the human liver model with competition for healthy subjects (red solid line; $FADH_2$ concentration of 0.43 μM), patient 1 (dotted blue line; $FADH_2$ concentration 0.6 μM) and patient 2 (dashed blue line; $FADH_2$ concentration 0.73 μM).

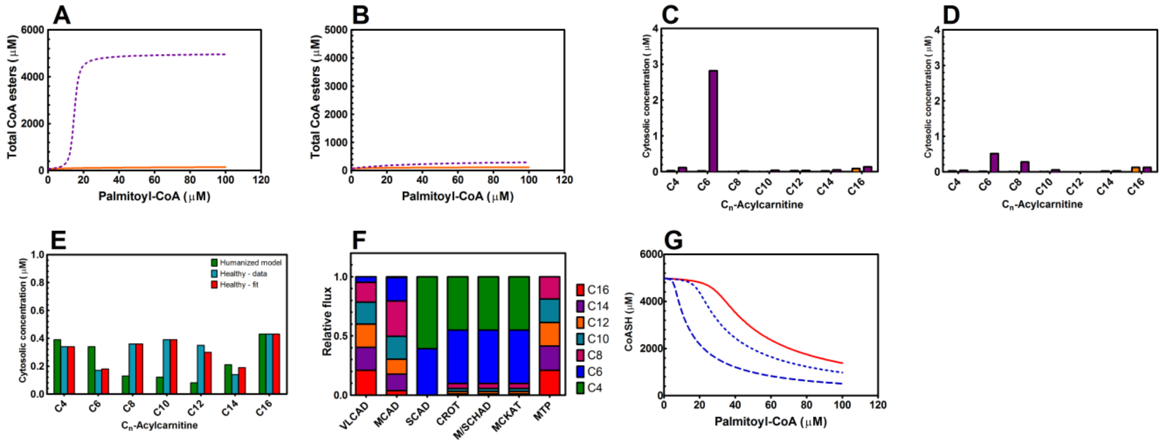

Supplement: Additional file 7: Figure S7. — Steady-state simulation data of the mouse and human liver mFAO. (PDF 243 kb) [file 12915_2016_327_MOESM7_ESM.pdf]
