# Supplementary material for: Living on the edge: substrate competition explains loss of robustness in mitochondrial fatty-acid oxidation disorders
Source: BMC Biol. 2016 Dec 7;14:107. doi: 10.1186/s12915-016-0327-5 (PMC5142382; doi:10.1186/s12915-016-0327-5)
Supplement: Additional file 11: Table S11. — List of primers used for the real-time PCR. (PDF 555 kb) [file 12915_2016_327_MOESM11_ESM.pdf]

Supplemental Table S11:

List of primers used for the real-time PCR

gene name	gene name complete	Locus	Forward primer sequence 5'-3'	Reverse primer sequence 5'-3'	TaqMan Probe sequence 5'-3'
**Cpt1a**	**carnitine palmitoyltransferase 1a**	**NM_013495.1**	CTC AGT GGG AGC GAC TCT TCA	GGC CTC TGT GGT ACA CGA CAA	CCT GGG GAG GAG ACA GAC ACC ATC CAA C
**Cpt1b**	**carnitine palmitoyltransferase 1b**	**NM_009948 / NM_013200**	CCA GAT GGA GAG GAT GTT CAA CA	AGA AGC GAC CTT TGT GGT AGA CA	CCC AGG CAA AGA GAC AGA CTT GCT ACA GC
**Cpt2**	**carnitine palmitoyltransferase 2**	**NM_009949**	CAG TGC ACA GAA GCC TCT CTT G	CTT CCC AAT GCC GTT CTC AA	CAC AAC ACT TCT GTC TTC CTG AAC TGG CTG
**SCAD**	**Short-chain acyl-CoA dehydrogenase**	**NM_007383**	ATGGTGACAAAATCGGCTGTT	CCGGGCAGTGGTGGAA	CCCTCAGTGAGCCAGGCAATGGC
**MCAD**	**Medium-chain acyl-CoA dehydrogenase**	**NM_007382**	GCA GCC AAT GAT GTG TGC TTA C	CAC CCT TCT TCT CTG CTT TGG T	CCC TCC GCA GGC TCT GAT GTG G
**LCAD**	**Long-chain acyl-CoA dehydrogenase**	**NM_007381 / NM_012819**	TAC GGC ACA AAA GAA CAG ATC G	CAG GCT CTG TCA TGG CTA TGG	CAC TTG CCC GCC GTC ATC TGG
**VLCAD**	**Very-long chain acyl-CoA dehydrogenase**	**NM_017366**	TCCTCAACAACGGAAGATTCG	GATCAACCGCCTTGGCAAT	CCTCGCAGGCACCATGAAATCCC
**CROT**	**Crotonase**	**NM_053119.2**	GTGAACTTGCCATGATGTGTGA	GGTCCCCAGGAGGATTTCTG	TCATCTATGCTGGCGAGAAAGCCCA
**M/SCHAD**	**Medium/short-chain hydroxyacyl-CoA dehydrogenase**	**NM_008212.4**	TGAAGCTGAAGAACGAGCTGTT	GTGATCTGCAAAGAAGACGTGTTG	CTGGACAAGTTCGCTGCAGAACACACC
**MTPα**	**Mitochondrial trifunctional protein subunit alpha**	**NM_178878.2**	CCAAGAAGCAACACGAATATCACA	GCATGCTATGGCAAGCTCAA	AAACTTGAAAAGTCCCCAAAGCCCGTTG
