# Supplementary material for: Living on the edge: substrate competition explains loss of robustness in mitochondrial fatty-acid oxidation disorders
Source: BMC Biol. 2016 Dec 7;14:107. doi: 10.1186/s12915-016-0327-5 (PMC5142382; doi:10.1186/s12915-016-0327-5)
Supplement: Additional file 12: Table S12. — List of peptides used for targeted proteomics of mFAO proteins. (PDF 146 kb) [file 12915_2016_327_MOESM12_ESM.pdf]

Supplemental Table S12:

List of peptides used for targeted proteomics of mFAO proteins

Protein	Peptide sequence
CPT1a	VWLYHDGR
CPT1b	ALLHGNCYNR
	ALADDVELYCFQFLPFGK
CPT2	QYGQTVATYESCSTAAFK
	SEYNDQLTR
SCAD	LVIAGHLLR
	ITEIYEGTSEIQR
MCAD	ANWYFLLAR
LCAD	THICVTR
VLCAD	IFEGANDILR
	FFEEVNDPAK
CROT	ISAQDAK
	AQFGQPEILLGTIPGAGGTQR
M/SCHAD	LVEVIK
	LGAGYPMGPFELLDYVGLDTTK
MCKAT	VGVPTETGALTLNR
	AALSAGK
MTPα	DGPGFYTTR
	THINYGVK
MTPβ	DQLLLGPTYATPK
	LAAAFAVSR
