# Supplementary material for: Living on the edge: substrate competition explains loss of robustness in mitochondrial fatty-acid oxidation disorders
Source: BMC Biol. 2016 Dec 7;14:107. doi: 10.1186/s12915-016-0327-5 (PMC5142382; doi:10.1186/s12915-016-0327-5)
Supplement: Additional file 13: Table S13. — Parameters for which we incorporated a human value to humanize the rat model. (PDF 87 kb) [file 12915_2016_327_MOESM13_ESM.pdf]

Supplemental Table S13

Parameters for which we incorporated a human value to humanize the rat model.

Parameter	Value
Vvlcad	0.019
Vmcad	0.01
Vscad	0.043
KmvlcadC16AcylCoAMAT	14
KmvlcadC14AcylCoAMAT	10
KmvlcadC12AcylCoAMAT	7
KmvlcadC10AcylCoAMAT	10
KmvlcadC8AcylCoAMAT	8
KmvlcadC6AcylCoAMAT	29
SfvlcadC16	0.51
SfvlcadC14	0.57
SfvlcadC12	1.0
SfvlcadC10	1.0
SfvlcadC8	0.64
SfvlcadC6	0.54
KmmcadC16AcylCoAMAT	23.8
KmmcadC14AcylCoAMAT	10
KmmcadC12AcylCoAMAT	9.3
KmmcadC10AcylCoAMAT	9.1
KmmcadC8AcylCoAMAT	8
KmmcadC6AcylCoAMAT	21.6
KmmcadC4AcylCoAMAT	71.4
SfmcadC16	0.15
SfmcadC14	0.24
SfmcadC12	0.50
SfmcadC10	0.54
SfmcadC8	0.86
SfmcadC6	1.0
SfmcadC4	0.31
KmscadC6AcylCoAMAT	33.9
KmscadC4AcylCoAMAT	12.9
SfscadC6	0.79
SfscadC4	1.0
