# Supplementary material for: Living on the edge: substrate competition explains loss of robustness in mitochondrial fatty-acid oxidation disorders
Source: BMC Biol. 2016 Dec 7;14:107. doi: 10.1186/s12915-016-0327-5 (PMC5142382; doi:10.1186/s12915-016-0327-5)
Supplement: Additional file 14: Table S14. — Estimated parameters for the human model. (PDF 102 kb) [file 12915_2016_327_MOESM14_ESM.pdf]

Supplemental Table S14

Estimated parameters for the human model.

List of the parameters with lower and upper boundaries, which were estimated when the model was fitted to the experimental data. The set of parameters was chosen based on a sensitivity analysis. The set contains the parameters for which the acylcarnitine concentrations were most sensitive.

Parameter	Original value	Lower boundary	Upper boundary	Estimated value
Vcpt1	0.012	0.0024	0.06	0.011
Kmcpt1C16AcylCoACYT	13.8	2.76	69.0	17.67
Kmcpt1CarCYT	250	50.0	1250	279.9
Vcact	0.42	0.084	2.10	2.10
Vcpt2	0.391	0.078	1.96	1.96
Vvlcad	0.019	0.0038	0.095	0.019
Vmcad	0.01	0.0020	0.050	0.013
Vscad	0.043	0.0086	0.22	0.10
KmvlcadC16AcylCoAMAT	14	7.00	28.0	8.72
KmvlcadC14AcylCoAMAT	10	5.00	20.0	5.00
KmvlcadC12AcylCoAMAT	7	3.50	14.0	14.00
KmvlcadC10AcylCoAMAT	10	5.00	20.0	18.75
KmvlcadC8AcylCoAMAT	8	4.00	16.0	12.12
KmvlcadC6AcylCoAMAT	29	14.5	58.0	14.50
KmvlcadFAD	0.12	0.024	0.60	0.10
KmmcadC16AcylCoAMAT	23.8	11.9	47.6	24.39
KmmcadC14AcylCoAMAT	10	5.00	20.0	5.00
KmmcadC12AcylCoAMAT	9.3	4.65	18.6	18.60
KmmcadC10AcylCoAMAT	9.1	4.55	18.2	16.71
KmmcadC8AcylCoAMAT	8	4.00	16.0	15.76
KmmcadC6AcylCoAMAT	21.6	10.8	43.2	10.80
KmmcadC4AcylCoAMAT	71.4	35.7	143	143
KmmcadFAD	0.12	0.024	0.60	0.10
KmscadC6AcylCoAMAT	33.9	17.0	67.8	17.0
KmscadC4AcylCoAMAT	12.9	6.45	25.8	18.15
KmscadFAD	0.12	0.024	0.60	0.10
Vcrot	3.6	0.72	18	0.72
Vmschad	1	0.20	5.00	0.27
Vmckat	0.377	0.075	1.89	0.39
KmmckatAcetylCoAMAT	30	6.00	150	68.11
KmmckatC16AcylCoAMAT	13.83	2.77	69.2	16.41
KmmckatC14AcylCoAMAT	13.83	2.77	69.2	16.92
KmmckatC12AcylCoAMAT	13.83	2.77	69.2	29.88
KmmckatC10AcylCoAMAT	13.83	2.77	69.2	25.37
KmmckatC8AcylCoAMAT	13.83	2.77	69.2	23.47
KmmckatC6AcylCoAMAT	13.83	2.77	69.2	15.48
KmmckatC4AcylCoAMAT	13.83	2.77	69.2	16.11
Vmtp	2.84	0.57	1.89	0.99
K1fadhsink	0.46	1.00E-6	0.77	0.43
K1nadhsink	16	1.00E-6	250	15.35
K1acesink	70	1.00E-6	5000	21.14
