# Supplementary material for: Living on the edge: substrate competition explains loss of robustness in mitochondrial fatty-acid oxidation disorders
Source: BMC Biol. 2016 Dec 7;14:107. doi: 10.1186/s12915-016-0327-5 (PMC5142382; doi:10.1186/s12915-016-0327-5)
Supplement: Additional file 15: Table S15. — Steady-state characteristics of the computer models. (PDF 93 kb) [file 12915_2016_327_MOESM15_ESM.pdf]

Supplemental Table S15

Steady-state characteristics of the computer models

Boundary metabolite concentrations, steady-state acylcarnitine concentrations in the cytosol, CoASH concentration in the mitochondrial matrix and NADH production rate (equal to the mFAO rate) of the mouse and human liver mFAO model with competition after parameter estimation.

	Mouse	Human	Unit
Boundary metabolite concentrations			
Malonyl-CoA in cytosol	0	0	µM
Carnitine in cytosol	200	200	µM
CoA in cytosol	140	140	µM
Total CoA in mitochondria	5000	5000	µM
Total FAD in mitochondria	0.77	0.77	µM
$FADH_2$	0.46	0.43	µM
Total NAD in mitochondria	250	250	µM
NADH	16	15.35	µM
Acetyl-CoA	70	21.14	µM
Concentrations in steady state			
C16AcylcarCYT	0.14	0.43	µM
C14AcylcarCYT	0.05	0.19	µM
C12AcylcarCYT	0.03	0.30	µM
C10AcylcarCYT	0.01	0.39	µM
C8AcylcarCYT	0.01	0.36	µM
C6AcylcarCYT	0.06	0.18	µM
C4AcylcarCYT	0.06	0.34	µM
CoASH	4888	4658	µM
Flux in steady state			
vnadhsink	0.008	0.006	$\mu mol.min^{-1}.mgMitoProtein^{-1}$
