# Supplementary material for: Living on the edge: substrate competition explains loss of robustness in mitochondrial fatty-acid oxidation disorders
Source: BMC Biol. 2016 Dec 7;14:107. doi: 10.1186/s12915-016-0327-5 (PMC5142382; doi:10.1186/s12915-016-0327-5)
Supplement: Additional file 16: Table S16. — Partition factor of the mouse and human model of mFAO. (PDF 100 kb) [file 12915_2016_327_MOESM16_ESM.pdf]

Supplemental Table S16

Partition factor of the mouse and human model of mFAO

	Mouse wild type	Mouse MCAD-KO	Human
CPT2			
C16	0.4	0.4	0.4
C14	0.1	0.1	0.1
C12	0.1	0.1	0.1
C10	0.1	0.1	0.1
C8	0.1	0.1	0.1
C6	0.1	0.1	0.1
C4	0.1	0.1	0.1
VLCAD			
C16	0.07	0.09	0.21
C14	0.48	0.47	0.19
C12	0.45	0.44	0.20
C10			0.19
C8			0.17
C6			0.05
LCAD			
C16	0.34	0.22	
C14	0.28	0.14	
C12	0.28	0.15	
C10	0.08	0.24	
C8	0.02	0.24	
MCAD			
C16			0.04
C14			0.14
C12	0.003		0.13
C10	0.29		0.19
C8	0.34		0.30
C6	0.36		0.20
C4	0.01		0.01
SCAD			
C6	0.03	0.5	0.39
C4	0.97	0.5	0.61
CROT; M/SCHAD; MCKAT			
C14	0.001	0.003	0.01
C12	0.002	0.005	0.02
C10	0.002	0.006	0.03
C8	0.004	0.01	0.04
C6	0.50	0.49	0.45
C4	0.50	0.49	0.45
MTP			
C16	0.20	0.20	0.21
C14	0.20	0.20	0.21
C12	0.20	0.20	0.20
C10	0.20	0.20	0.20
C8	0.20	0.20	0.19
